# Evaluation of an App-Delivered Psychological Flexibility Skill Training Intervention for Medical Student Burnout and Well-being: Randomized Controlled Trial

**DOI:** 10.2196/42566

**Published:** 2023-02-06

**Authors:** Elizabeth Ditton, Brendon Knott, Nicolette Hodyl, Graeme Horton, Christopher Oldmeadow, Frederick Rohan Walker, Michael Nilsson

**Affiliations:** 1 Centre for Rehab Innovations University of Newcastle Callaghan Australia; 2 Hunter Medical Research Institute New Lambton Heights Australia; 3 College of Health, Medicine and Wellbeing University of Newcastle Callaghan Australia; 4 Contextual Interventions Newcastle Australia; 5 New South Wales Regional Health Partners Newcastle Australia; 6 Lee Kong Chian School of Medicine Nanyang Technological University Singapore Singapore

**Keywords:** burnout, psychological, burnout interventions, well-being, medicine, medical student, digital intervention, app-delivered intervention, individualized intervention, randomized controlled trial, RCT, randomized, Acceptance and Commitment Training, stress, mobile health, mHealth, mobile phone

## Abstract

**Background:**

Physician burnout is a common problem, with onset frequently occurring during undergraduate education. Early intervention strategies that train medical students in psychological flexibility skills could support well-being and mitigate burnout risks associated with unmodifiable career stressors. There is a need for randomized controlled trials to assess effectiveness. As psychological flexibility varies contextually and among individuals, tailoring interventions may improve outcomes. Smartphone apps can facilitate individualization and accessibility, and the evaluation of this approach is an identified research priority.

**Objective:**

This study aimed to evaluate the effectiveness of a stand-alone app–delivered Acceptance and Commitment Training intervention for improving medical students’ self-reported burnout, well-being, psychological flexibility, and psychological distress outcomes. We aimed to explore whether an individualized app would demonstrate benefits over a nonindividualized version.

**Methods:**

This parallel randomized controlled trial was conducted with a sample of medical students from 2 Australian universities (N=143). Participants were randomly allocated to 1 of 3 intervention arms (individualized, nonindividualized, and waitlist) using a 1:1:1 allocation ratio. Individualized and nonindividualized participants were blinded to group allocation. The 5-week intervention included an introductory module (stage 1) and on-demand access to short skill training activities (stage 2), which students accessed at their own pace. Stage 2 was either nonindividualized or individualized to meet students’ identified psychological flexibility training needs.

**Results:**

The mean differences in change from baseline between the intervention groups and the waitlist group were not statistically significant for burnout outcomes: exhaustion (primary; individualized: −0.52, 95% CI −3.70 to 2.65, *P*=.75; nonindividualized: 1.60, 95% CI −1.84 to 5.03, *P*=.37), cynicism (individualized: −1.26, 95% CI −4.46 to 1.94, *P*=.44; nonindividualized: 1.00, 95% CI −2.45 to 4.46, *P*=.57), and academic efficacy (individualized: 0.94, 95% CI −0.90 to 2.79, *P*=.32; nonindividualized: 2.02, 95% CI 0.02-4.03, *P*=.05). Following the intervention, the individualized group demonstrated improved psychological flexibility (0.50, 95% CI 0.12-0.89; *P*=.01), reduced inflexibility (0.48, 95% CI −0.92 to −0.04; *P*=.04), and reduced stress (−6.89, 95% CI −12.01 to 5.99; *P*=.01), and the nonindividualized group demonstrated improved well-being (6.46, 95% CI 0.49-12.42; *P*=.04) and stress (−6.36, 95% CI −11.90 to −0.83; *P*=.03) compared with waitlist participants. Between-group differences for the individualized and nonindividualized arms were not statistically significant. High attrition (75/143, 52.4%) was observed.

**Conclusions:**

This trial provides early support for the potential benefits of Acceptance and Commitment Training for medical student well-being and psychological outcomes and demonstrates that psychological flexibility and inflexibility can be trained using a smartphone app. Although postintervention burnout outcomes were not statistically significant, improvements in secondary outcomes could indicate early risk mitigation. Replication studies with larger samples and longer-term follow-up are required, and future research should focus on improving implementation frameworks to increase engagement and optimize individualization methods.

**Trial Registration:**

Australian New Zealand Clinical Trials Registry 12621000911897; https://tinyurl.com/2p92cwrw

**International Registered Report Identifier (IRRID):**

RR2-10.2196/32992

## Introduction

### Burnout and the Medical Profession

The medical profession is facing a burnout crisis [1]. Prevalence estimates among physicians range between 37% [2] and 80% [3], and the enduring challenges associated with the COVID-19 pandemic have exacerbated this existing problem [4,5]. Burnout is a psychological outcome of exposure to work-related stress characterized by varying degrees of exhaustion (feelings of overload and depleted emotional energy), cynicism (detachment, withdrawal behaviors, and diminished idealism), and inefficacy (the perception that one’s own work performance lacks quality and value) [6,7]. Although previously believed to emerge during the later stages of a medical career [8], a high global prevalence among medical students (44.2% [9]) is indicative of earlier onset. Increasingly, burnout is arising in response to pervasive imbalances between medical education stressors and a student’s coping resources, and this can persist throughout an individual’s professional life [10,11]. Individuals affected by burnout are at risk of a range of associated outcomes that extend beyond the work context, including poor physical [6,12] and psychological health (including diminished well-being [13], depression [14,15], anxiety [16], and suicide [14,17]). Furthermore, burnout impedes medical service delivery and productivity through its adverse impact on work engagement (eg, reduced participation) [18] and performance (eg, increased medical errors and diminished quality of patient care [2,15]).

Developing effective strategies to address the problem of physician burnout and its associated outcomes was an identified research priority before the pandemic [1,19], with a particular emphasis on early intervention strategies that could facilitate prevention [1,20-22]. However, progress toward this agenda was disrupted by the emergence of COVID-19, which required the medical profession to rapidly prioritize and respond to the ensuing public health crisis, often under conditions of uncertainty and insufficient resources [4]. The persistent stress of the pandemic has contributed to an increase in rates of physician burnout [23] and declining mental health among medical students, to the extent that many report reconsidering their decision to pursue a medical career [24]. There are growing concerns that the potential endurance of these adverse mental health impacts beyond the pandemic could further diminish the resilience of the medical workforce and health care systems, adding to the urgency of identifying and deploying effective interventions [4].

### Adaptive Psychological Skill Training for Medical Students

Organizational interventions that modify external stressors within the work or study environment (eg, inadequate resources, excessive workloads, and time pressures) have demonstrated benefits [25-27] and are essential to burnout prevention strategies [28]. However, the pandemic has highlighted that the modifiability of some of these factors may be limited during extended periods of crisis when health care resources are stretched beyond capacity. Medical students in a recent study reported that pandemic-related stressors contributed to elevated burnout and diminished mental health, some of which were less modifiable under the circumstances (eg, web-based learning fatigue, restricted opportunities for clinical experience, and mandatory isolation) than others (eg, quality of web-based learning) [24,29]. Furthermore, physicians and medical students encounter unmodifiable demands inherent to their training and work, resulting in unavoidable contact with certain risk factors during the normal course of their careers [8] (eg, academic pressures, exposure to death and dying [11,30], and role responsibility [15]).

Psychological and behavioral responses to demands and stressors play a role in burnout development among medical students, whose risk of burnout is almost doubled when maladaptive coping patterns are adopted [30-33]. There is recent evidence suggesting that, in addition to its augmentation of external stressors, the pandemic has adversely affected medical students’ psychological resources for coping with these challenges [24]. Maladaptive coping patterns are unlikely to spontaneously improve over time without intervention [34], which may contribute to the increasing risk of burnout that medical students face as they progress through training and into their careers [8,30,35]. Individual-level interventions have the potential to buffer against burnout and other psychological ill health outcomes by training *modifiable* cognitive, emotional, and behavioral skills that can facilitate adaptive responses to unmodifiable contextual stressors [8,34,36,37]. There is growing support for the implementation of such interventions during undergraduate medical training, which is recognized as a critical stage of learning and career preparation [21,38,39]. Physicians experiencing burnout have proposed that learning self-care should be prioritized equally with clinical skills during medical education [40]. By assisting students in developing adaptive coping repertoires, individual-level interventions can pre-emptively prepare them to respond to the inevitable stressors of medical education and future practice in ways that support their psychological health and well-being [21,38,39]. This early intervention strategy could offer longer-term benefits for burnout prevention within the medical profession [8,30,31,37,38].

### Intervention Model: Psychological Flexibility

Despite the considerable increase in the number of studies investigating such interventions in recent years, systematic reviews have highlighted the need for more rigorous studies identifying which adaptive psychological skill sets can be trained to produce optimal improvements in burnout and well-being outcomes among medical students [21,28]. Psychological flexibility is a set of adaptive cognitive and behavioral skills that is a promising intervention target [36,41-43]. The psychological flexibility model encompasses 6 modifiable flexibility (and corresponding *inflexibility*) processes: present-moment awareness (nonawareness of present moment), experiential acceptance (experiential avoidance), cognitive defusion (cognitive fusion), self-as-context (self-as-content), contact with values (lack of contact with values), and committed action toward values (inaction) [44,45]. When faced with challenging situations or uncomfortable internal experiences, individuals who are high in psychological flexibility tend to respond in ways that are effective in the moment and supportive of their broader well-being [46,47], including bringing conscious awareness and openness to the conditions of their present-moment experiences and purposefully committing to effective actions that align with personally held values [44]. Conversely, psychological inflexibility manifests as behavioral rigidity in response to internal (eg, thoughts, emotions, and physical sensations) experiences, which can have an adverse impact on an individual’s capacity to adapt and function in psychologically healthy ways [44]. An individual can learn to improve their psychological flexibility skills using a range of intervention approaches, including Acceptance and Commitment Training (ACT) [48,49]. Rather than focusing on directly modifying psychological symptoms, ACT aims to increase psychological flexibility and decrease psychological inflexibility processes in the service of expanding adaptive, values-based behavioral repertoires [44]. This can alter the way in which an individual responds to stressful experiences, producing secondary benefits across a wide range of psychological and organizational variables [49-51].

Within the broader literature, psychological flexibility is associated with well-being [52,53] and has been shown to protect against burnout and adverse mental health outcomes in stressful situations [42,47,52] (including depression and anxiety [51]). Interventions that train individuals to develop and strengthen their psychological flexibility skills are effective in reducing work-related stress and burnout severity [36,41,54-57] and improving well-being [36,41,43]. Mediation studies demonstrate that ACT interventions exert their beneficial impacts on burnout and well-being by improving an individual’s psychological flexibility [36], suggesting that this skill set is a mechanism of change for these outcomes. In a longitudinal ACT intervention study, improvements in psychological flexibility mediated reductions in exhaustion [42], and this prevented the later development of cynicism [42]. This is an important finding with respect to the potential burnout prevention benefits of psychological flexibility training among medical students, for whom exhaustion is the most prevalent factor and is considered a foundational manifestation of burnout [2,9].

There is early evidence suggesting that psychological flexibility skills may function as important personal resources in the medical profession [31]. Low psychological flexibility is associated with burnout risk among medical students, physicians [58], and resident physicians [59]. Medical students who report low psychological flexibility also demonstrate diminished satisfaction with life and greater personal distress when seeing others in harm, which may increase burnout risk during their careers [31]. Furthermore, burnout risk is higher among medical students who engage in experiential avoidance [11,32] or non–values-based actions [60]. Conversely, higher psychological flexibility was recently found to predict lower burnout among medical students and physicians during the pandemic [58]. There is minimal research examining the benefits of psychological flexibility skill training interventions for medical students. A recent study found that distressed medical students who completed ACT training as a requirement of their undergraduate medical curriculum demonstrated improved burnout outcomes, but this study did not include a control group [61]. A small semiexperimental study demonstrated improvements in well-being and psychological distress among female medical students in Iran following completion of an ACT intervention [62]. Further rigorous efficacy studies are needed to evaluate whether training medical students in psychological flexibility skills improves burnout and psychological well-being outcomes.

### App-Based Intervention Delivery

#### Accessibility

A recent systematic review of individual resource-building interventions for medical students highlighted the need for research evaluating the effectiveness of nontraditional delivery methods such as smartphone apps [21]. As medical students use smartphones frequently [63], stand-alone app–delivered interventions have the potential for cost-effective scalability [39,64] and can offset known accessibility barriers by providing anonymous and private access to medical students concerned about mental health stigma [11,32,64]. Stand-alone apps offer accessibility in times when face-to-face delivery is not an option, such as during pandemic-related lockdowns [65], and can deliver brief training components at convenient times for those with busy schedules [64,66]. Psychological skill generalization may also be enhanced because of the accessibility of training opportunities in everyday life situations [67]. However, feasibility trials of app-based psychological interventions for medical students also indicate that maintaining engagement in this accessible medium can be challenging [63,68].

#### Individualization

App technologies have been identified as important to advance key research priorities within the psychological flexibility literature because of their potential to facilitate methodologies that accommodate individual heterogeneity and enhance intervention precision [69]. Although the benefits of ACT have been assessed in >1000 randomized controlled trials (RCTs) [50], traditional approaches evaluating the effectiveness of generic interventions by comparing aggregated group data are limiting as they overlook important information regarding individual differences in training needs and outcomes [69-71]. Generalized deployment of interventions found to be effective at the group level is likely to result in some individuals not receiving the type or amount of training they need and others receiving more than necessary [57,70]. With respect to psychological flexibility processes, research suggests that individuals might require training in different skills at different times and in different situations [45]. Although high psychological flexibility is often associated with low psychological inflexibility (and vice versa), several distinct and more complex profiles have recently been observed [46], indicating that flexibility and inflexibility processes do not necessarily exist at opposite ends of a spectrum but may vary temporally and contextually within individuals [45,46,72]. Thus, there is a growing focus on individualized approaches in which intervention decisions are driven by an individual participant’s identified needs and skill deficits in varying moments and contexts [69,70]. Apps have the potential to facilitate both the real-time ecological momentary assessment (EMA) [69] of these needs in a participant’s everyday life and the targeted delivery of just-in-time adaptive intervention components that align with these needs [73]. Importantly, as an individualized approach adjusts for heterogeneity at the intervention level, an RCT study design can be used to assess its treatment utility over a nonindividualized approach [70].

As ACT is a theory-driven intervention with practical training components that have clear functional links to the corresponding core process within the psychological flexibility model [44,48,74], it is well suited to individualized delivery via an app [75]. Levin et al [75] demonstrated a simple EMA strategy for identifying a participant’s present-moment psychological flexibility training needs, involving a brief subjective assessment of which process an individual felt they were experiencing the most difficulty with each time they accessed an app-delivered ACT intervention. This information was used to individualize the training by delivering a practical ACT skill activity that aligned with the identified psychological flexibility process at that moment. Using a 3-arm RCT, the researchers demonstrated that, compared with a nonindividualized ACT app and a nontreatment group, university students who engaged with the individualized app demonstrated statistically significantly greater improvements in psychological distress and well-being. Empirical evaluation of individualized ACT interventions is in its early stages, and more research is needed to examine effectiveness and advance the development of individualization methodologies [69,75,76]. To date, no studies have examined the potential burnout and well-being benefits of training medical students in psychological flexibility skills using individualized or nonindividualized ACT apps.

### Study Aims

The aim of this RCT was to evaluate the effectiveness of an app-delivered ACT intervention for medical students with respect to burnout (exhaustion [primary outcome], cynicism, and academic efficacy), well-being, and psychological flexibility and inflexibility outcomes. We hypothesized that medical students who engaged with either an individualized or nonindividualized version of the ACT app would demonstrate greater postintervention improvements in outcomes than those in a waitlist group and that intervention effects would be greater for the individualized group than for the nonindividualized group. We aimed to examine whether any observed postintervention improvements in burnout or well-being would be mediated by improvements in psychological flexibility and inflexibility. Furthermore, we aimed to explore whether engaging in either version of the ACT app would improve other relevant secondary psychological outcomes (stress, depression, and anxiety).

## Methods

### Ethics Approval

Ethics approval for this study was granted by the University of Newcastle Human Research Ethics Committee on January 21, 2021 (approval ID: H-2020-0311), and ratified by the University of New England Human Research Ethics Committee on February 11, 2021.

### Trial Design

This study was a 3-arm, parallel RCT of a 2-stage psychological flexibility skill training app for medical student burnout and well-being. Randomization was performed within the app using a 1:1:1 allocation ratio and a simple randomization procedure where each student had a 1 in 3 chance of allocation to each intervention arm (individualized intervention, nonindividualized intervention, and waiting list). The inclusion of the nonindividualized group was an important element of this research design as it provided a generic ACT intervention control condition against which to evaluate the potential relative benefits (or “treatment utility” [70]) of the individualized approach adopted [75]. The functional limitations of the app meant that it was not possible to stratify randomization by participant baseline characteristics. Allocation was blinded for participants assigned to the individualized and nonindividualized intervention groups. Blinding was not possible for the waitlist group.

### Recruitment and Study Setting

**Figure 1:**
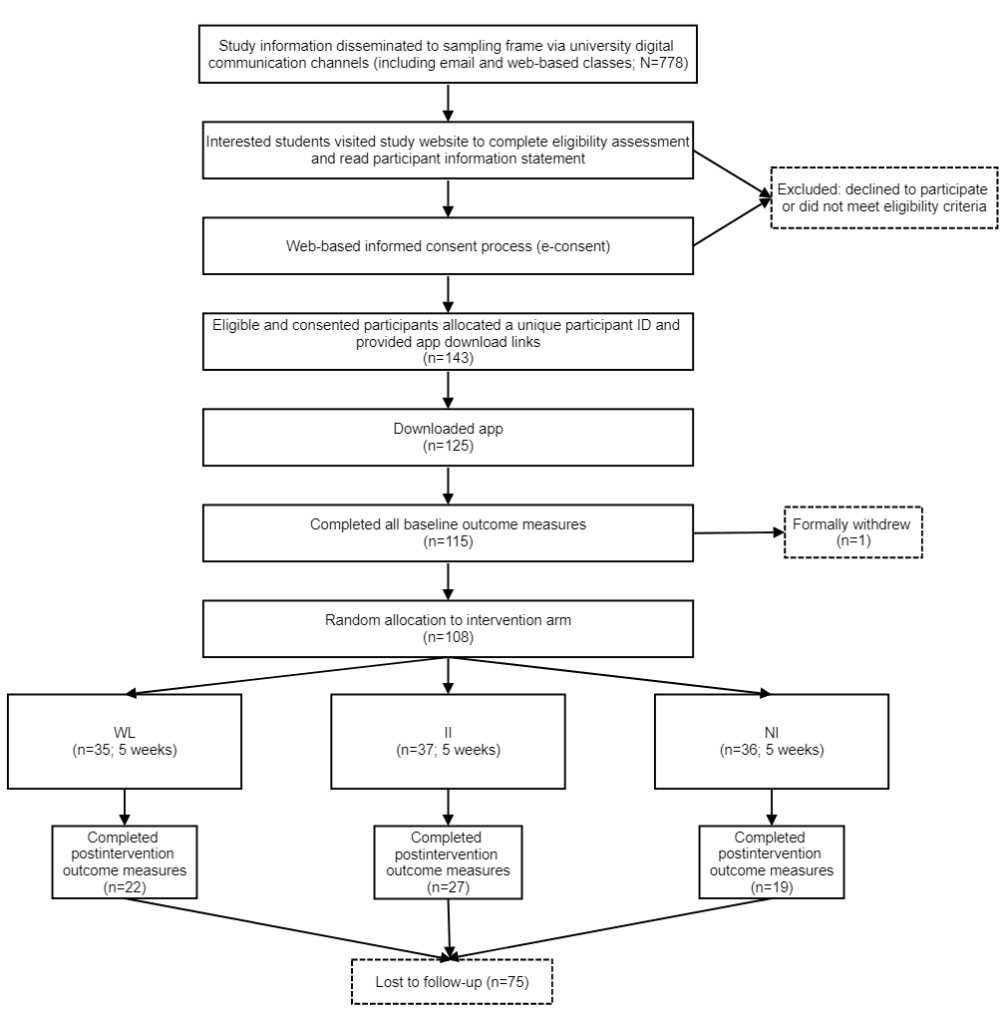
Participant flow diagram. II: individualized intervention; NI: nonindividualized intervention; WL: waiting list.

Recruitment was conducted for 6 weeks during August 2021 and September 2021 (refer to Figure 1 for the participant flow diagram). This unintentionally coincided with a mandated lockdown because of a regional COVID-19 outbreak. The sampling frame was students enrolled in first, second, fourth, and fifth years of the Joint Medical Program (JMP) at the University of Newcastle or the University of New England, Australia (N=778). During the first and second years of the JMP, students predominantly engage in in-class academic learning, whereas the fourth and fifth years involve a stronger focus on applied clinical training. Students enrolled in the third year of the JMP were not invited to participate in this study as they had previously taken part in a feasibility trial of the app.

Students were invited to participate in a trial to assess the efficacy of a psychological flexibility skill training app for well-being and burnout prevention. An invitation email was sent to students’ university accounts from a JMP administrative account approximately every 2 weeks during the recruitment period, and a member of the research team also delivered a verbal invitation at the end of web-based classes. Students were provided with a URL and QR code that gave them access to the enrollment website (located on a secure web-based survey and database platform, REDCap [Research Electronic Data Capture; Vanderbilt University] [77,78], hosted at Hunter Medical Research Institute) during the recruitment period.

Students who accessed the enrollment website were assessed for eligibility and were eligible for inclusion if they had regular access to a reliable internet connection and an electronic device compatible with app use (smartphone or tablet). There were no ineligibility criteria. Participation was voluntary, confidential, and independent of students’ academic programs. Students were required to read a participant information statement before deciding whether to enroll in the study. This included information about the purpose of the study, time required to complete outcome measures (approximately 10 minutes at each time point), intervention and study flow, privacy and data storage, and withdrawal processes. Students who elected to participate registered their consent through an e-consent process. Following completion of a brief self-report demographic assessment (baseline participant characteristics), enrolled students were provided with links to download the app via the Play Store (Android) and App Store (Apple). The app was password-protected to ensure that only study participants had access to the outcome measures (closed surveys) and intervention.

### Data Privacy

To ensure the privacy and anonymity of participant data, 2 password-protected databases were used to store personal and demographic information (in an identifiable “Participant Information Database”) separately from the outcome data (stored in an anonymous “Study Database”). Outcome data were deidentified, with participant ID used as a linkage key. Only the lead author had access to the participant information database. Members of the research team did not have access to identifying participant information. To protect privacy while using the app, students input their unique participant ID as their username when registering and accessing their account.

### Procedures

#### Overview

The study period for each participant commenced when they downloaded the app and registered their account. All further assessment, intervention, and data collection procedures were conducted within the app, which students accessed independently during the study period. The intervention and assessment components were resumable, allowing students to engage with the app at their own pace. Participants first completed baseline psychological outcome measures followed by a brief (10 minutes) onboarding session, which outlined how to use the app and introduced psychological flexibility as an adaptive behavioral skill set that could help students do what is important to them while effectively navigating life’s challenges. Students were then randomized into 1 of the 3 intervention arms, after which the app was programmed to deliver the intervention pathway associated with their group allocation. The individualized and nonindividualized groups were given access to the psychological flexibility training intervention via the app for 5 weeks. For students assigned to the waiting list, access to app content was disabled for 5 weeks. After the study period, all 3 groups were asked to complete the postintervention outcome measures. Upon completion, waitlist participants were given access to the individualized version of the app, and intervention group participants were given continued access to their allocated version. All students who completed the postintervention outcome measures were given an Aus $30 (US $20.70) digital gift voucher to acknowledge the time commitment involved in participation.

The 5-week intervention duration was set in consultation with the participating medical schools. This provided students with the opportunity to participate in the study during the semester while reducing participation burden by ensuring that the timing of outcome measures did not correspond with critical assessment periods. This duration was considered appropriate as Levin et al [75] observed intervention effects for a similar 4-week program, and feasibility trialing of the current app demonstrated that medical students engaged most frequently during the early weeks of access [68].

#### Intervention

##### Overview

The intervention was an ACT-based psychological flexibility [44] training program delivered via a smartphone app (“BiSi: Build it. Sustain it.”) created for this study. A more detailed protocol for this study is available elsewhere [79]. BiSi was developed by clinical psychologists (ED and BK) with extensive experience in the psychological flexibility model and involved the adaptation of existing ACT concepts and training activities to suit the app-based context and target participant group. The intervention was delivered in 2 stages.

##### Stage 1: Learn the Concepts (Introductory Module)

Stage 1 involved the delivery of an introductory module (“Learn the Concepts”), which was identical for the individualized and nonindividualized groups. The purpose of the module was to familiarize students with the psychological flexibility model and its potential benefits and provide a conceptual framework for understanding the stage 2 experiential skill training activities. Students could complete the module over multiple sittings (total completion time of approximately 60 minutes depending on individual pace). The module comprised 7 sections (<10 min each) presented in a fixed order. Students were required to complete each section before progressing to the next. Section 1 provided psychoeducation about burnout (focusing on destigmatizing burnout-related experiences and how to recognize the signs) and well-being (including the importance of choosing actions that involve the consideration of personal well-being). Sections 2 to 7 provided education about each psychological flexibility and inflexibility process and outlined how psychological flexibility skills could be implemented to support well-being and protect against burnout. Although the module was primarily conceptual, experiential components encouraged self-reflection and provided opportunities to practice each skill set (eg, personal values identification, mindfulness, experiential acceptance, thought defusion, perspective shifting, and values-based goal setting). Written and audio versions of the psychoeducational content were provided, along with accompanying images. Some skill activities were presented in either written or audio format depending on which translated best to the app-based delivery mode. Completion of the stage 1 introductory module unlocked students’ access to the stage 2 on-demand skill-training dashboard.

##### Stage 2: Learn the Behaviors (On-Demand Skill Training)

Stage 2 provided access to a library of short (3 to 8 minutes) experiential psychological flexibility skill activities that students could practice at any time (on demand). Each of the 6 psychological flexibility processes had its own dashboard where all ACT training activities for that skill set were displayed on a list (20 activities per process; 120 in total). Although most activities were presented in audio format with accompanying images, some included written components and items that required the user to input a response.

During stage 2, students from both intervention groups were presented with a main dashboard (home screen) each time they opened the app. To access the psychological flexibility skill activities, students selected “Practice a Skill” on the home screen and were presented with a single-item EMA screening question (“Which of the following are you having the most difficulty with today?”) to identify which psychological flexibility skill set might be most relevant to their training needs on that occasion (adapted from Levin et al [75])*.* Students selected 1 of 6 response options, each of which corresponded to one of the 6 psychological flexibility processes: “Struggling with your feelings*”* (acceptance), “Unable to do what matters to you” (committed action), “Stuck in your thoughts” (defusion), “Stuck in autopilot or struggling to stay in the present moment” (present-moment awareness), “Disconnected from a sense of meaning or purpose” (values), and “Stuck in stories about who you are or who you should be” (self-as-context).

The EMA screening question was used to tailor the intervention to the training needs of students in the individualized group. Upon completion of the screening question, participants in the individualized group were presented with the dashboard corresponding to the psychological flexibility skill set they had identified as having the most difficulty with on that occasion (eg, if a student reported feeling *disconnected from a sense of meaning or purpose*, the app displayed the values dashboard). This allowed students in the individualized group to practice a skill targeted to the area of identified need each time they accessed the app. For the nonindividualized group, students’ responses to the screening question had no impact on the skill set in which they received training. After responding to the screening question, students in the nonindividualized group were presented with 1 of the 6 psychological flexibility dashboards at random (simple randomization; each process had an equal chance of selection). Students were informed that there were 2 intervention groups and that the only difference was the way in which skill activities were selected for them by the app.

Once students were given access to a psychological flexibility dashboard, they could practice any activity from that skill set, either by choosing from the list or by allowing the app to select for them. The latter involved a simple randomization process where each activity had an equal chance of selection (1 in 20). Upon completion of an activity, students had the option to complete another within the same psychological flexibility skill set. If they selected “yes,” these steps were repeated until the participant elected to discontinue.

The version of BiSi implemented in this study incorporated medical student feedback from a small feasibility trial of the app [68] with the aim of enhancing intervention relevance and engagement. This included clear explanations of what to expect during each stage of the intervention, providing progress indicators for all app components, providing earlier opportunities for personally relevant experiential learning and self-reflection (during stage 1), and delivering content in both written and audio formats where practical (during stage 1). We also introduced positive reinforcement components that were delivered after students completed certain activities or a certain number of activities (eg, achievement badges and experience points). Students were asked to complete a minimum of 4 stage 2 skill activities but were also encouraged to optimize skill learning by practicing more regularly. Students were sent reminders to use the app at 6 PM each day. This frequency and time were based on feedback from the feasibility trial. Although reminders were intended to be delivered using push notifications as per medical students’ reported preferences, this function did not operate as intended during the study, and reminders were sent by email instead.

### Outcome Measures

#### Psychological Outcome Measures

##### Overview

Self-report psychological outcome measures were administered at 2 time points: baseline (collected before randomization) and postintervention measurement (collected 5 weeks after randomization). The outcome measures were presented in the order of the following sections. Participants were required to provide a response to each item before progressing to the next. Owing to the limitations of the app, participants could not alter their responses once submitted.

##### Burnout

Exhaustion was selected as the primary outcome for this study as it is the most prevalent factor among medical students [9] and physicians [2] and early improvements in exhaustion following an ACT intervention may prevent the future development of other burnout factors (cynicism) [42]. Cynicism and academic efficacy were assessed as secondary burnout outcomes. The Maslach Burnout Inventory [80] is a valid [81], gold-standard [82] measure of the 3-factor burnout model. Of the versions available, the General Survey for Students (MBI-GS [S]) [80,81] was the most appropriate for the medical student cohort. The 16-item self-report questionnaire assesses the degree to which students are experiencing each factor using a 7-point Likert scale ranging from 0 (“never”) to 6 (“every day”). Higher total scores for exhaustion and cynicism and lower total scores for academic efficacy are indicative of higher frequencies of burnout-related experiences. The reliability of the MBI-GS (S) has been demonstrated among medical students [66,83]. In this study, internal consistency was excellent for exhaustion (Cronbach α=.90) and good for cynicism (Cronbach α=.83) and academic efficacy (Cronbach α=.83). Items were presented in consecutive order, with 1 item displayed per app screen (18 screens, including introductory text).

##### Well-being

Well-being was measured as a secondary outcome using the Mental Health Continuum–Short Form [84], which assesses self-reported hedonic (ie, feeling good) and eudaimonic (ie, functioning well) aspects of well-being [85]. Participants rate the frequency of 14 well-being experiences during the previous month using a 6-point Likert scale ranging from 0 (“never”) to 5 (“every day”). Total well-being is estimated by summing all scale items. Higher scores reflect higher overall well-being. The scale has demonstrated validity [84,85], reliability [85,86], and sensitivity to change in web-based intervention studies [87]. Internal consistency was excellent in our sample (Cronbach α=.92). Items were presented in consecutive order, with 1 item displayed per app screen (15 screens, including introductory text).

##### Psychological Flexibility and Inflexibility

The Multidimensional Psychological Flexibility Inventory–Short Form [45] was administered to evaluate whether the intervention improved medical students’ psychological flexibility and reduced their psychological inflexibility (secondary outcomes). This 24-item self-report questionnaire assesses the frequency of psychological flexibility and psychological inflexibility experiences during the previous 2 weeks using a 6-point Likert scale ranging from 1 (“never true”) to 6 (“always true”). The scale provides separate average global composite scores for psychological flexibility and inflexibility as recent research suggests that they are related but “conceptually distinct” processes that may have disparate relationships with well-being and psychological distress outcomes and may respond differently to interventions [45,46,72]. The scale has demonstrated validity [72,88] and reliability [88,89] and is responsive to changes over time [45]. This study demonstrated good internal consistency for psychological flexibility (Cronbach α=.87) and inflexibility (Cronbach α=.87). Items were presented in consecutive order, with 1 item displayed per app screen (24 screens).

##### Psychological Distress (Depression, Anxiety, and Stress)

The Depression, Anxiety, and Stress scale–21 [90] is a valid and reliable [90,91] 21-item self-report questionnaire assessed using a 4-point Likert scale. The measure provides subscale scores that estimate the severity of depression, anxiety, and stress symptoms [90]. We included this measure to explore whether the ACT intervention improved these secondary outcomes and evaluate whether these psychological distress factors affected engagement in the intervention, as has been observed in previous digital intervention studies [92]. Internal consistency was excellent for depression (Cronbach α=.90) and good for anxiety (Cronbach α=.81) and stress (Cronbach α=.87) in this study. Items were presented in consecutive order, with 7 items displayed per app screen (3 screens).

#### Intervention Engagement Outcomes

Behavioral engagement data and subjective intervention feedback were collected from participants throughout the study.

##### Study Attrition

Attrition was defined as formal withdrawal or loss to follow-up at any stage during the study without completing the postintervention outcome measures.

##### Intervention Adherence

Adherence to the individualized and nonindividualized intervention arms was defined as the completion of all stage 1 components and engagement in at least 4 skill activities during stage 2. This level of adherence provided students with the opportunity to learn about and practice each of the psychological flexibility processes (stage 1) and practice the skills a few times in their everyday lives (stage 2).

##### Intervention Feedback

Students were invited to submit feedback on their experience of using the app via a form presented halfway through stage 1. The feedback form was also accessible via the main dashboard. During stage 2, participants rated whether they liked each skill activity they practiced using a single-item binary measure (*thumbs up* [like] or *thumbs down* [dislike] icon) [93]. Participants were invited to report concerns or harms experienced during the study using contact links provided within the app and via email communication (eg, daily reminders).

### Data Analysis

#### Power Analysis

R (statistical computing package; R Foundation for Statistical Computing) was used to calculate the standard power for a 2-tailed independent-sample *t* test for between-group differences not accounting for repeated measures. This was considered a conservative choice as reliable within-person correlation information needed for power based on a mixed model was not available. This analysis indicated that a sample of 117 participants would provide sufficient power (80%) to detect a clinically meaningful effect size (SD 0.65) between either intervention arm and the control arm in the primary outcome (exhaustion), measured using the MBI-GS (S), with a type-I error rate of 5%. We aimed to recruit up to 153 participants to provide a 30% margin for attrition.

#### Participant Psychological Characteristics at Baseline

Average participant psychological characteristics at baseline were compared with reference samples (including other medical student or general population samples from previously published studies) using single-sample *t* tests.

#### Intervention Effects

This study adopted an intention-to-treat analysis, which included data collected from all participants randomized into a study group. Each participant’s data were analyzed based on the study group to which they were randomized irrespective of their degree of intervention engagement or whether they met the adherence criteria. This approach maintains randomization benefits and minimizes bias when assessing intervention efficacy [94].

Differences between the intervention arms were assessed using linear mixed regression models for primary (exhaustion) and secondary (cynicism, academic efficacy, well-being, psychological flexibility and inflexibility, depression, anxiety, and stress) outcomes. A separate model was estimated for each outcome variable. The models included fixed categorical effects for time (baseline as the referent), intervention group, the interaction between intervention and time, and self-reported baseline participant characteristics that were imbalanced after randomization (ie, gender and whether students were studying medicine as their first career [“first career”]). The model included a random participant-specific intercept to account for the repeated measures for each participant. The adjusted difference between the intervention groups in mean change from baseline to postintervention measurement is presented, as well as the within-group change from baseline, 95% CIs, and 2-tailed *P* values. Model assumptions were assessed by inspecting residual plots. For outcomes that violated modeling assumptions (ie, demonstrated nonnormality of residuals or nonconstant variance), a robust linear mixed-effects model was estimated, with the same fixed and random effects as the previous models.

To control for elevated type-I errors arising from having 2 primary contrasts of interest (each intervention vs control), we followed a hierarchical testing procedure for the primary outcome (exhaustion) where the less intensive intervention (nonindividualized) would only be declared to be significantly different from the control (at a 5% significance threshold) if the more intensive intervention (individualized) was statistically significantly different from the control at a 5% significance level. All other analyses were exploratory. We note that the study was not powered to directly compare the individualized and nonindividualized intervention arms.

#### Mediation Analyses

We planned to conduct mediation analyses to assess whether changes in process outcomes (psychological flexibility and inflexibility) between baseline and postintervention measurement mediated changes in psychological outcomes. However, the observed intervention effects did not support the implementation of these analyses for reasons outlined in the Results section.

#### Study Attrition, Intervention Adherence, and Engagement

Baseline participant demographic and psychological characteristics were compared between (1) those who were lost to follow-up at any point during the study versus those who were not and (2) those who met intervention adherence criteria versus those who did not using chi-square tests for categorical variables (or the Fisher exact test where cell size was <5) and independent-sample Student *t* tests for continuous variables (Welch *t* tests used where the assumption of equal variance was violated). The average rates of engagement during stage 2 were compared between the individualized and nonindividualized groups using independent-sample *t* tests.

## Results

### Participant Demographics

A total of 143 medical students were enrolled in this study. Table 1 shows demographic characteristics of the total enrolled sample and each intervention group. The enrolled participants were aged between 18 and 51 years (mean 24.0, SD 5.48 years), and more than half (88/143, 61.5%) were female. Most participants were nonindigenous (133/143, 93%) and domestic students (135/143, 94.4%) and were training in medicine as their first career (108/143, 75.5%). The average time spent in the workforce was 5.74 (SD 5.71) years. A substantial proportion of the enrolled students (122/143, 85.3%) reported having previously experienced burnout, and 21% (30/143) were engaging in psychological treatment at the time of the study. Students rated the quality of their health, diet, and self-care using a Likert scale ranging from 1 (“very poor”) to 5 (“excellent”). Mean ratings for quality of health (mean 3.72, SD 0.89) and diet (mean 3.61, SD 0.73) fell between “average” (3) and “good” (4), whereas mean ratings for self-care (mean 3.19, SD 0.82) were closer to an “average” rating.

**Table 1:** Participant demographic characteristics at baseline (by study group allocation and total; N=143).

			II^a^ (n=37)		NI^b^ (n=36^c^)		WL^d^ (n=35)		Not allocated (n=35)		Total enrolled
Age (years), mean (SD; range)			23.7 (5.60; 18-46)		25.4 (5.78; 19-42)		22.1 (3.01; 18-31)		24.7 (6.55; 19-51)		24.0 (5.48; 18-51)
**Gender, n (%)**											
	Female	26 (70.3)		17 (47.2)		23 (65.7)		22 (62.9)		88 (61.5)	
	Male	11 (29.7)		18 (50)		9 (25.7)		13 (37.1)		51 (35.7)	
	Nonbinary	0 (0)		0 (0)		3 (8.6)		0 (0)		3 (2.1)	
**Enrollment, n (%)**											
	Domestic	35 (94.6)		34 (94.4)		32 (91.4)		34 (97.1)		135 (94.4)	
	International	2 (5.4)		1 (2.8)		3 (8.6)		1 (2.9)		7 (4.9)	
**Year of study, n (%)**											
	1	9 (24.3)		6 (16.7)		11 (31.4)		18 (51.4)		44 (30.8)	
	2	15 (40.5)		14 (38.9)		7 (20)		7 (20)		43 (30.1)	
	4	7 (18.9)		7 (19.4)		8 (22.9)		4 (11.4)		26 (18.2)	
	5	6 (16.2)		8 (22.2)		9 (25.7)		6 (17.1)		29 (20.3)	
Indigenous, n (%)			2 (5.4)		4 (11.1)		0 (0)		4 (11.4)		10 (7)
First career, n (%)			26 (70.3)		25 (69.4)		32 (91.4)		25 (71.4)		108 (75.5)
Years in the workforce, mean (SD)			5.19 (6.0)		6.54 (5.95)		3.91 (3.12)		7.34 (6.69)		5.74 (5.71)
Previous burnout, n (%)			34 (91.9)		31 (86.1)		27 (77.1)		30 (85.7)		122 (85.3)
Current therapy, n (%)			6 (16.2)		7 (19.4)		11 (31.4)		6 (17.1)		30 (21)
Health rating, mean (SD)			3.62 (0.92)		4.00 (0.80)		3.40 (0.98)		3.83 (0.86)		3.72 (0.89)
Diet rating, mean (SD)			3.43 (0.87)		3.97 (0.57)		3.43 (0.71)		3.54 (0.82)		3.61 (0.73)
Self-care rating, mean (SD)			3.22 (0.81)		3.29 (0.66)		3.17 (0.71)		3.06 (0.84)		3.19 (0.82)

^a^II: individualized intervention.

^b^NI: nonindividualized intervention.

^c^Demographic data missing for 1 participant in this group.

^d^WL: waiting list.

### Participant Psychological Characteristics at Baseline

Outcome scores were calculated for all participants who completed the measures at baseline and only those who were randomized to an intervention arm. Removal of the participants lost to follow-up before random allocation did not greatly alter the mean scores for any psychological outcome. Therefore, we compared the average psychological characteristics of only the participants who proceeded to randomization and were included in the efficacy analyses (108/143, 75.5%) with reference samples (other medical students or the general population) from previously published studies (Multimedia Appendix 1 [66,88,90,95]).

On average, medical students presented a mixed profile of burnout scores, demonstrating exhaustion levels (mean 16.29, SD 7.20) comparable with those of a sample of medical students not experiencing burnout (mean 14.96, SD 5.71; *t*_269_=1.69; *P=*.09), academic efficacy scores (mean 24.69, SD 6.47) comparable with those of medical students who perceived themselves to be high in burnout (mean 24.81, SD 5.35; *t*_216_=0.15; *P=*.88), and cynicism scores (mean 10.87, SD 6.92) significantly lower than those of the burnout sample (mean 14.44, SD 5.59; *t*_216_=4.19; *P<*.001) but higher than those of the nonburnout sample (mean 7.59, SD 5.16; *t*_269_=4.46; *P<*.001) [66]. Total well-being scores (mean 43.03, SD 13.31) were significantly lower than those of a large multi-institutional sample of medical students in the United States (mean 47.0, SD 12.67; *t*_2796_=3.12; *P=*.002) [95]. Psychological inflexibility scores (mean 3.21, SD 0.91) were significantly higher than those of a general population sample (mean 2.73, SD 0.90; *t*_2769_=5.43; *P<*.001) [88]. Average depression (mean 14.89, SD 10.25), anxiety (mean 8.50, SD 8.09), and stress (mean 11.87, SD 10.34) scores for the medical student sample in this study were significantly higher than the general population averages (*depression:* mean 6.34, SD 6.97, *t*_3020_=12.27, and *P<*.001; *anxiety:* mean 4.7, SD 4.91, *t*_3020_=7.67, and *P<*.001; *stress:* mean 10.11, SD 7.91, *t*_3020_=2.24, and *P=*.03) [90].

### Intervention Effects

#### Overview

Table 2 shows the results for the burnout; well-being; psychological flexibility and inflexibility; and depression, anxiety, and stress outcomes.

**Table 2:** Data summaries of outcomes at each time point (baseline and postintervention measurement) for each treatment arm (mean and SD), mixed model–based estimates of within-group change (95% CI), and estimated between- and within-group change (95% CI and *P* value).

Outcome, subcategory, and intervention group				Baseline (n=108), mean (SD)		Postintervention measurement (n=68), mean (SD)		Baseline–postintervention measurement estimated change (95% CI)		*P* value
**Burnout**										
	**Exhaustion (primary)**									
		WL^a^	17.91 (7.02)		17.05 (6.81)		−1.61 (−3.96 to 0.745)		N/A^b^	
		II^c^	16.65 (7.22)		14.48 (6.85)		−2.13 (−4.27 to 0.008)		N/A	
		NI^d^	14.33 (7.09)		14.79 (5.60)		−0.01 (−2.51 to 2.49)		N/A	
		II-WL	N/A		N/A		−0.52 (−3.70 to 2.65)		.75	
		NI-WL	N/A		N/A		1.60 (−1.84 to 5.03)		.37	
		II-NI	N/A		N/A		−2.12 (−5.41 to 1.17)		.21	
	**Cynicism**									
		WL	11.5 (7.35)		12.0 (7.62)		−0.59 (−1.94 to 0.77)		N/A	
		II	11.7 (7.01)		9.74 (5.70)		−1.76 (−3.91 to 0.39)		N/A	
		NI	9.36 (6.32)		9.95 (5.54)		0.50 (−2.01 to 3.02)		N/A	
		II-WL	N/A		N/A		−1.26 (−4.46 to 1.94)		.44	
		NI-WL	N/A		N/A		1.00 (−2.45 to 4.46)		.57	
		II-NI	N/A		N/A		−2.26 (−5.57 to 1.05)		.18	
	**Academic efficacy**									
		WL	23.2 (6.29)		23.9 (6.27)		1.29 (0.51 to 2.08)		N/A	
		II	25.9 (6.42)		27.5 (5.29)		1.25 (0.01 to 2.49)		N/A	
		NI	24.9 (6.60)		26.4 (6.86)		2.33 (0.86 to 3.80)		N/A	
		II-WL	N/A		N/A		0.94 (−0.90 to 2.79)		.32	
		NI-WL	N/A		N/A		2.02 (0.02 to 4.03)		.05	
		II-NI	N/A		N/A		−1.08 (−3.00 to 0.84)		.27	
**Well-being**										
	**Total well-being**									
		WL	39.5 (13.3)		40.4 (13.1)		3.83 (1.49 to 6.18)		N/A	
		II	44.2 (13.7)		48.1 (11.3)		3.51 (−0.20 to 7.22)		N/A	
		NI	45.2 (12.5)		52.1 (8.8)		7.22 (2.87 to 11.57)		N/A	
		II-WL	N/A		N/A		2.74 (−2.77 to 8.26)		.33	
		NI-WL	N/A		N/A		6.46 (0.49 to 12.42)		.04	
		II-NI	N/A		N/A		−3.71 (−9.43 to 2.00)		.21	
**Psychological flexibility**										
	**Flexibility**									
		WL	3.65 (0.73)		3.69 (0.79)		0.26 (0.09 to 0.42)		N/A	
		II	3.74 (0.87)		4.22 (0.83)		0.48 (0.22 to 0.74)		N/A	
		NI	3.78 (0.73)		4.10 (0.72)		0.32 (0.02 to 0.62)		N/A	
		II-WL	N/A		N/A		0.50 (0.12 to 0.89)		.01	
		NI-WL	N/A		N/A		0.35 (−0.07 to 0.76)		.11	
		II-NI	N/A		N/A		0.16 (−0.24 to 0.56)		.44	
	**Inflexibility**									
		WL	3.32 (0.91)		3.34 (0.84)		−0.28 (−0.47 to −0.10)		N/A	
		II	3.27 (1.04)		2.77 (0.69)		−0.53 (−0.82 to −0.23)		N/A	
		NI	3.03 (0.76)		2.83 (0.75)		−0.28 (−0.62 to 0.07)		N/A	
		II-WL	N/A		N/A		−0.48 (−0.92 to −0.04)		.04	
		NI-WL	N/A		N/A		−0.23 (−0.70 to 0.24)		.34	
		II-NI	N/A		N/A		−0.25 (−0.70 to 0.21)		.29	
**Psychological distress**										
	**Depression^e^**									
		WL	11.8 (9.49)		11.7 (8.92)		−0.54 (−3.28 to 2.19)		N/A	
		II	12.8 (12.1)		7.7 (7.25)		−4.40 (−6.93 to −1.86)		N/A	
		NI	11.1 (9.4)		6.42 (5.56)		−3.95 (−6.92 to −0.97)		N/A	
		II-WL	N/A		N/A		−3.85 (−7.58 to −0.12)		.046	
		NI-WL	N/A		N/A		−3.40 (−7.44 to 0.64)		.10	
		II-NI	N/A		N/A		−0.45 (−4.36 to 3.46)		.82	
	**Anxiety^e^**									
		WL	8.63 (7.46)		7.13 (6.29)		−1.20 (−3.19 to 0.79)		N/A	
		II	9.41 (9.3)		6.44 (7.28)		−3.10 (−4.94 to −1.26)		N/A	
		NI	7.61 (7.5)		4.00 (3.83)		−3.45 (−5.62 to −1.29)		N/A	
		II-WL	N/A		N/A		−1.90 (−4.61 to 0.81)		.17	
		NI-WL	N/A		N/A		−2.25 (−5.19 to 0.69)		.14	
		II-NI	N/A		N/A		0.36 (−2.49 to 3.20)		.81	
	**Stress^e^**									
		WL	13.9 (8.99)		15.8 (8.8)		2.24 (−1.51 to 5.99)		N/A	
		II	16.1 (11.30)		11.6 (8.16)		−4.65 (−8.14 to −1.15)		N/A	
		NI	14.5 (10.40)		10.0 (7.42)		−4.12 (−8.17 to −0.06)		N/A	
		II-WL	N/A		N/A		−6.89 (−12.01 to 5.99)		.01	
		NI-WL	N/A		N/A		−6.36 (−11.90 to −0.83)		.03	
		II-NI	N/A		N/A		−0.53 (−5.88 to 4.82)		.85	

^a^WL: waiting list.

^b^N/A: not applicable.

^c^II: individualized intervention.

^d^NI: nonindividualized intervention.

^e^Robust linear mixed-effects models estimated because of violation of linear mixed regression modeling assumptions (ie, nonnormality of residuals or nonconstant variance).

#### Intervention (Individualized and Nonindividualized) Versus Waitlist Group Comparisons

##### Burnout

There were no statistically significant differences between the intervention arms and the waitlist control group for burnout outcomes (exhaustion [primary outcome]: −0.52, 95% CI −3.70 to 2.65, and *P=*.75 for individualized and 1.60, 95% CI −1.84 to 5.03, and *P=*.34 for nonindividualized; cynicism: −1.26, 95% CI −4.46 to 1.94, and *P=*.44 for individualized and 1.00, 95% CI −2.45 to 4.46, and *P=*.57 for nonindividualized; academic efficacy: −0.90, 95% CI −0.90 to 2.79, and *P=*.32 for individualized and 2.02, 95% CI 0.02-4.03, and *P=*.05 for nonindividualized).

##### Well-being

The estimated change in total well-being between baseline and postintervention measurement was significantly greater for the nonindividualized group (0.52) than for the waitlist group (0.27; *P=*.04), indicating that medical students in the nonindividualized group experienced improved well-being following intervention engagement compared with students who received no intervention.

##### Psychological Flexibility and Inflexibility

**Figure 2:**
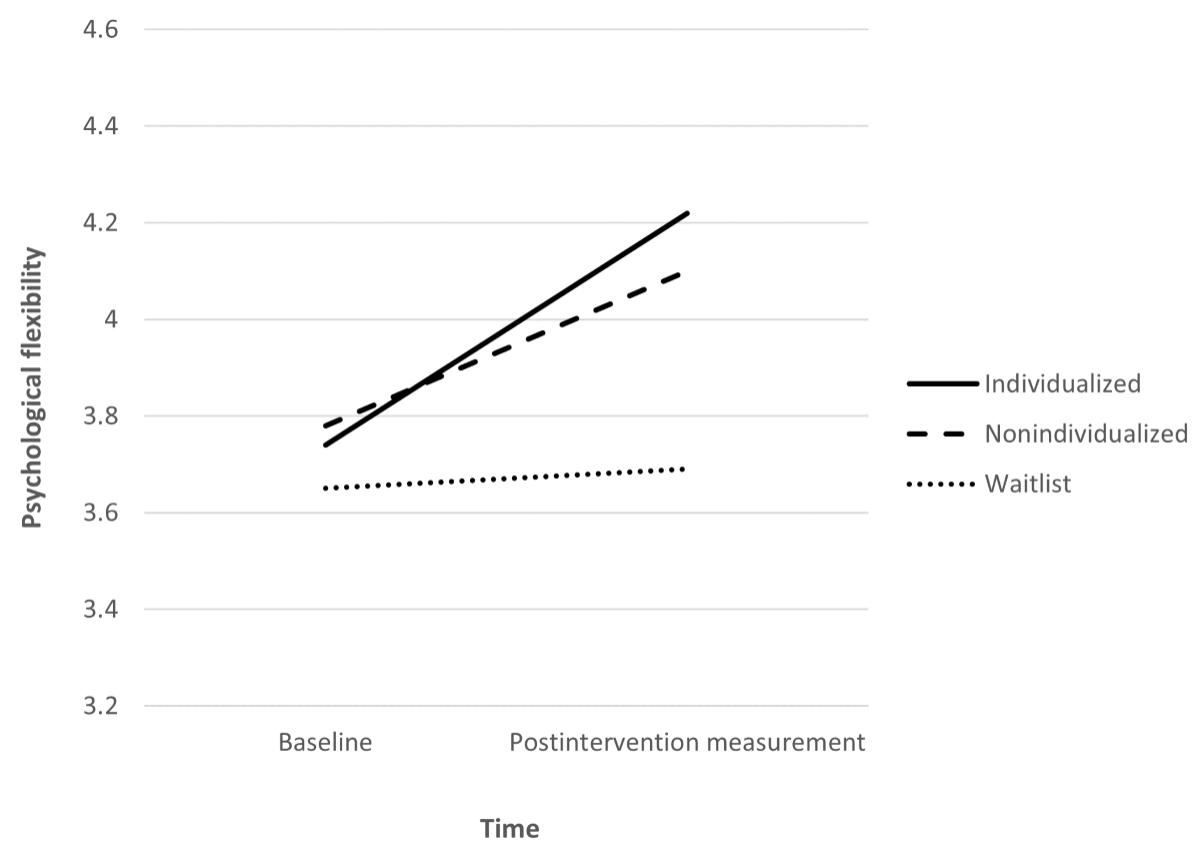
Mean psychological flexibility scores by intervention group at baseline and postintervention measurement.

The estimated increase in psychological flexibility between baseline and postintervention measurement was 0.50 points greater for the individualized group than for the waitlist group (0.26), and this difference was significant (*P=*.01). Similarly, the estimated decrease in psychological inflexibility between baseline and postintervention measurement was significantly greater for the individualized group (−0.53) than for the waitlist group (−0.28; *P=*.04). These findings indicate that medical students in the individualized group experienced improvements in both psychological flexibility (Figure 2) and psychological inflexibility following engagement in the intervention compared with students who received no intervention.

##### Depression

The estimated change in depression between baseline and postintervention measurement was significantly greater for the individualized group (−4.40) than for the waitlist group (−0.54; *P=*.046). This indicates that depressive symptoms reduced significantly more for students who engaged in the individualized version of the app than for those who received no intervention.

##### Anxiety

There were no statistically significant differences between the intervention arms and the control group for anxiety.

##### Stress

**Figure 3:**
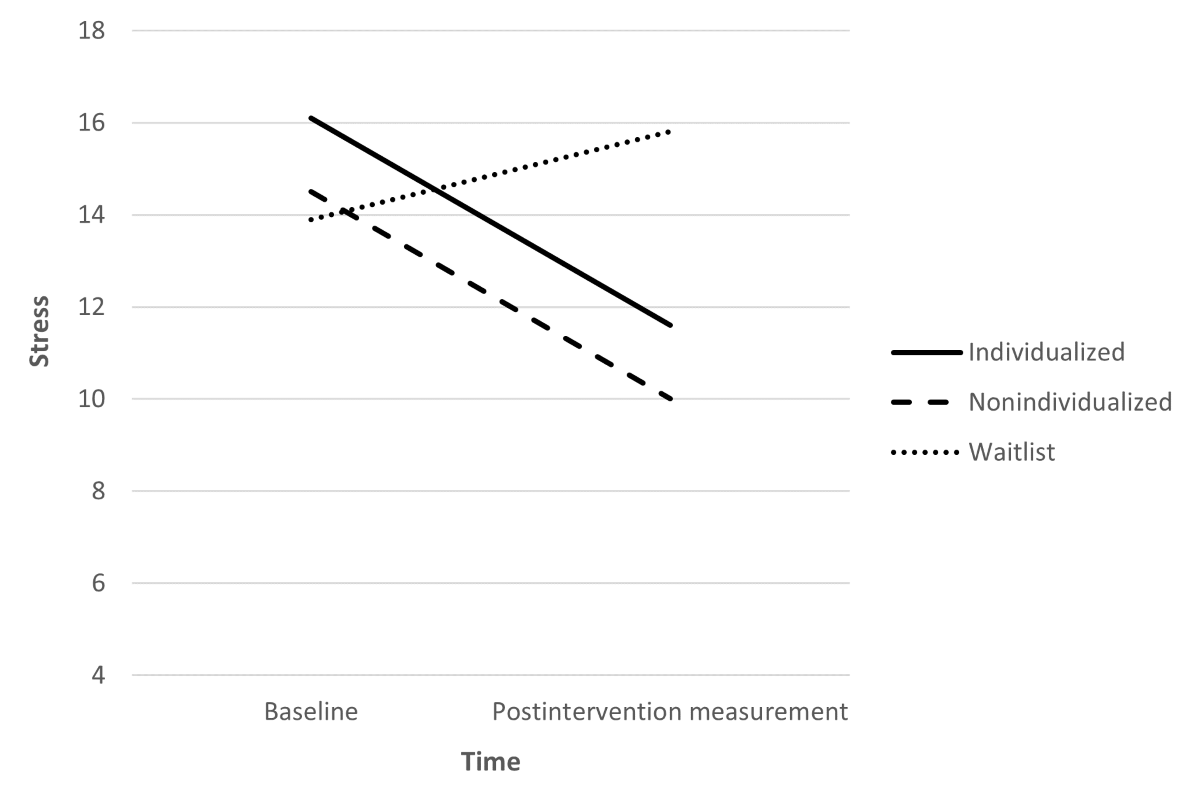
Mean stress scores by intervention group at baseline and postintervention measurement.

There was a significant difference in the estimated change in stress between baseline and postintervention measurement for both the individualized (*P=*.01; Figure 3) and nonindividualized (*P=*.03) groups compared with the waitlist group (individualized=−4.65; nonindividualized=−4.12; waiting list=2.24), indicating that stress reduced significantly more for participants in both intervention groups than for participants in the waitlist group.

#### Individualized Versus Nonindividualized Group Comparisons

There were no statistically significant differences between the individualized and nonindividualized arms in any of the psychological outcomes assessed.

### Mediation Analyses

Although the study was statistically powered to conduct psychological flexibility mediation analyses for the primary outcome (exhaustion), given the failure to observe a meaningful intervention effect for this outcome, formal mediation analyses of exhaustion and the secondary psychological outcomes would have been too exploratory to have any confidence in the results.

### Study Attrition, Intervention Adherence, and Engagement

A high rate of attrition (75/143, 52.4%) was observed during this study (Multimedia Appendix 2). Almost half (35/75, 47%) of those lost to follow-up discontinued before commencing the intervention and were not randomized to a study group. Only 1 participant formally withdrew, citing perceived personal irrelevance of the baseline outcome measures as the reason. Students lost to follow-up did not differ significantly from those who completed the study with respect to baseline demographic or psychological characteristics (Multimedia Appendix 3).

Of the participants allocated to either the individualized or nonindividualized group, 63% (46/73) completed stage 1, and 29% (21/73) met the predefined adherence criteria (ie, completion of at least 4 stage 2 activities; Multimedia Appendix 4). Intervention adherence rates did not differ by group allocation (N=73, *χ*^2^_1_=3.0; *P=*.08). The only baseline participant characteristics associated with adherence rates were *age*—students who adhered to the study were older on average (mean 27.5, SD 7.9 years) than those who did not (mean 23.3, SD 4.0 years; *t*_70_=−2.31; *P=*.03)—and *medicine as a first career*—significantly lower adherence rates among students studying medicine as a first career (10/51, 20%) than among those with previous career experience in another field (11/21, 52%; N=72, *χ*^2^_1_=7.7; *P=*.005; Multimedia Appendix 5).

Participants completed a total of 255 skill activities during stage 2, with those in the individualized group completing an average of 6 (SD 5.7) activities and those in the nonindividualized group completing an average of 8 (SD 12.4) activities. This difference was not significant (*t*_36_=−0.53; *P=*.60). Of note, 3 participants accounted for 38.8% (99/255) of all skill activities completed during stage 2. With these participants removed, the average number of activities completed was 5 (SD 3.8) for the individualized group and 3 (SD 2.1) for the nonindividualized group. This between-group difference was not significant (*t*_33_=1.69; *P=*.10).

### Intervention Harms and Feedback

No harms were reported during the study. Participants who used the in-app feedback form (15/143, 10.5%) reported finding the intervention content interesting and helpful in relation to their psychological health and well-being. Some reported that, despite finding the app helpful, time was a key barrier to engagement. Other feedback included requests for additional usability functions, such as dark mode and written versions of activities that were only presented in audio format. Students in the intervention groups liked 87.1% (222/255) of the skill activities completed during stage 2 and disliked 12.9% (33/255).

## Discussion

### Intervention Outcomes

This is the first RCT to evaluate individualized and nonindividualized versions of a smartphone app–delivered psychological flexibility skill training (ACT) intervention for medical students. Although we did not demonstrate immediate postintervention improvements in burnout outcomes among medical students who used the app, the observed improvements in psychological flexibility, well-being, and stress outcomes provide promising support for this intervention approach.

On the basis of the existing literature, we anticipated that training medical students in psychological flexibility skills using an app-delivered ACT intervention would lead to improvements in burnout. Primary (exhaustion) and secondary (cynicism and academic efficacy) burnout outcomes for the sample in this study were not significantly affected by engagement in either version of the ACT app at the end of the 5-week intervention access period. We selected exhaustion as the primary burnout outcome because of its high reported prevalence among medical students [9] and the possible longer-term burnout prevention benefits associated with this factor’s early responsivity to psychological flexibility interventions [42]. However, baseline exhaustion and cynicism scores for this sample were suggestive of low levels of burnout compared with reference medical student samples [66], which may have produced a floor effect with respect to the potential for improving these outcomes. Previous studies assessing the benefits of web-based ACT interventions were less likely to demonstrate intervention effects when the samples under investigation demonstrated nonclinical baseline levels of the psychological outcome under investigation [96].

Interestingly, medical students in this sample demonstrated high baseline levels of stress compared with a general population sample [90], and our exploratory secondary analyses showed significant postintervention improvements in this outcome for both the individualized and nonindividualized groups. As stress is a risk factor for burnout among medical students [63], it is possible that stress reduction might have been an early indicator of ACT intervention efficacy and that medical students may require longer to implement psychological flexibility skills in adaptive ways in their everyday lives before experiencing benefits for the more chronic and distal state of burnout. This is supported by the results of a recent RCT with a heterogeneous employee sample, which demonstrated that reduced stress was an immediate response to an 8-week web-based ACT intervention, whereas improvements in burnout became more evident 1 year later [36]. Similarly, systematic review findings indicate that mitigation of early stress experiences could be an important first step for ACT interventions with respect to improving burnout or preventing its development [97]. Future investigation of this theory regarding the current ACT app will require the prospective evaluation of stress as a primary outcome and replication of these study findings, as well as longer-term follow-up of burnout outcomes. This could provide greater clarity regarding the potential importance of psychological flexibility processes in facilitating adaptive responses to stress in medical students and the possible impact on burnout prevention.

As psychological flexibility is associated with well-being [52,53], we expected that engaging in an ACT intervention would improve this outcome among medical students. Medical students in the nonindividualized group experienced significant postintervention improvements in well-being compared with the waitlist group. To our knowledge, this is the first RCT to demonstrate the efficacy of an ACT intervention for medical student well-being and the first to show that well-being benefits can be achieved among medical students using an app-based mode of delivery. These findings suggest that providing medical students with access to ACT skill training interventions could support personal thriving and positive psychological health, which is a key objective of medical educators [38]. Importantly, previous research has shown that improvements in well-being can offset adverse consequences of burnout (eg, unprofessional behaviors and suicidal ideation) [95,98]. Furthermore, although traditional ACT interventions can require considerable time commitment [99-101], the observed well-being outcomes followed a relatively brief app-based intervention, which is an important consideration for this (and any other) time-poor cohort [64,66]. Future research is needed to replicate these secondary findings and explore whether continued intervention engagement is required to maintain well-being benefits in the long term.

It is unclear why only the nonindividualized group experienced significant improvements in well-being given that both versions of the ACT app delivered similar training content and that the individualized intervention was expected to produce stronger outcomes. This discrepancy is not attributable to engagement as there were no significant between-group differences in intervention adherence. Although we were unable to evaluate mediation effects for well-being outcomes among nonindividualized participants, we note that psychological flexibility and inflexibility did not improve significantly in this group. It is possible that unassessed mediating factors may have contributed to the observed inconsistencies [102], and future studies are required to evaluate this further to better understand the processes underlying the well-being benefits of this ACT-based app.

Following the intervention, medical students who engaged in the individualized version of the app demonstrated increased psychological flexibility and reduced psychological inflexibility. This is the first study to show that medical students can be trained to improve psychological flexibility and inflexibility using an individualized ACT intervention delivered via an app. Although there was no corresponding improvement in burnout and well-being outcomes for this intervention group, there is an extensive literature base demonstrating that this adaptive skill set is broadly beneficial to individual health and functioning [50] (including anxiety, depression, physical health outcomes, satisfaction with life, and work performance [47,72,103]). In fact, psychological flexibility has recently been described as “the cornerstone of psychological health and resiliency” [104]. Demonstrating that this skill set can be trained using an accessible digital delivery methodology and a low time commitment could be an important step toward developing interventions that can improve medical students’ psychological health and functioning more broadly. Our exploratory findings hinted at the potential wider benefits of improving medical students’ psychological flexibility, showing that depressive symptoms among those in the individualized group also improved following engagement in the intervention. Longer-term studies are needed to evaluate whether improving medical students’ psychological flexibility and inflexibility is beneficial to distal burnout outcomes or whether these processes might have greater relevance to other important psychological outcomes (eg, well-being, stress, and depression).

Although medical students who engaged in either the individualized or nonindividualized intervention demonstrated improvements in at least one psychological outcome compared with those in the waitlist group, unlike Levin et al [75], we did not observe statistically significant differences between the 2 intervention groups for any of the outcomes assessed. However, in addition to being underpowered to detect these effects, it is possible that methodological factors obfuscated our ability to evaluate the true potential treatment utility of individualized over nonindividualized psychological flexibility skill training. Although our individualization method was similar to that of Levin et al [75], students from both groups in this study were required to complete the same introductory module before progressing to their respective individualized or nonindividualized skill training. Our intention-to-treat analysis approach meant that students were evaluated based on the group they were allocated to rather than the degree to which they actually engaged in the intervention. Owing to the high level of nonadherence, both groups contained participants who either did not reach the point where the intervention methodologies diverged or did not engage sufficiently in the skill training stage for individualization to have a meaningful impact. Given this considerable overlap between the intervention groups and the likelihood that our study did not adequately differentiate between individualized and nonindividualized conditions, these nonsignificant findings should be interpreted cautiously. The refinement of individualized ACT interventions is important for progressing key goals within the psychological flexibility literature, including improving precision and effectiveness for all individuals who access training [69,70]. Furthermore, ensuring the efficiency of psychological interventions is particularly important for medical students because of the impact of time constraints on engagement [64,66]. Comparing differences in outcomes between an EMA-driven individualized app and a nonindividualized condition offers a number of advantages to future research, including the facilitation of empirical evaluation of whether individualized ACT interventions do strengthen outcomes, providing a study design framework that facilitates continued optimization of individualization methods and furthering our understanding of how specific intervention components drive changes in adaptive psychological processes [69,70]. Future app-based ACT research adopting this study design should ensure that individualized and nonindividualized interventions are appropriately distinct and that sample sizes are large enough to detect potential between-group differences.

### Adherence

Adherence to the app was an observed challenge in this early-phase study. Only two-thirds of the intervention group participants (46/73, 63%) completed the introductory module, fewer than one-third (21/73, 29%) met the adherence criteria, and overall study attrition was high. Low adherence has the potential to undermine the feasibility and scalability of this app-delivered intervention approach, and understanding the contributing factors is essential for effective development and implementation [63]. Unlike previous digital intervention studies [92], the baseline psychological characteristics of the medical student sample in this study did not significantly affect attrition or adherence to the ACT app. Older students and those who had previously worked in a career other than medicine were more likely to adhere, and it is possible that these students perceived the intervention to be more relevant or necessary because of previous life or work experiences. “Like” data from stage 2 suggest that medical students who remained engaged in this study were satisfied with the content of the ACT intervention, which may have motivated continued participation among these students. As immediate feedback for stage 1 activities was not collected, it is unclear whether early discontinuation might have been affected by satisfaction with the initial intervention content. However, the few students who used the available feedback forms during stage 1 reported favorable experiences regarding content but cited lack of time and usability factors as engagement and adherence barriers.

Although previous research suggests that medical student engagement in digital interventions may be improved by the incorporation of face-to-face training components [63], the stand-alone app–based mode of delivery was adopted because of uncertainty associated with COVID-19 restrictions at the time of development. Given the likelihood of ongoing accessibility barriers for psychological interventions among medical students [32] and the potential reach of this approach, future research should incorporate a formal evaluation of the user experiences contributing to engagement and disengagement [63] to develop stronger and more engaging implementation frameworks for stand-alone apps.

### Strengths and Limitations

This study had several strengths, including its randomized controlled design; assessment of a theory-driven intervention specifically developed for medical students; incorporation of medical student end-user feedback before implementation; evaluation among students from more than one university; and its app-based delivery, which facilitated access and implementation during a COVID-19 lockdown. Although we were underpowered to detect differences between the individualized and nonindividualized groups, the use of technologies that can adapt interventions to individual needs and the adoption of research methodologies that assess the treatment utility of these interventions are important to the psychological flexibility literature and should be explored further [69].

A strength of this study was the separate evaluation of psychological flexibility and inflexibility [45], which are often measured as opposite ends of a single-factor construct [105]. Although many individuals who are high in psychological flexibility demonstrate correspondingly low inflexibility, some individuals may show high levels of both simultaneously, whereas others show mixed patterns across all core processes [46]. Given that psychological flexibility and inflexibility appear to be conceptually distinct processes that may have disparate relationships with well-being and psychological distress outcomes and may respond differently to interventions [45,46,72], evaluating them separately provides a stronger understanding of intervention methodologies that can address this heterogeneity [70,71]. This demonstration of postintervention improvements in both psychological flexibility and inflexibility following engagement in the individualized ACT app provides promising support for this approach with respect to the simultaneous strengthening of adaptive behavioral repertoires and undermining of maladaptive repertoires among medical students.

Although we made the best efforts to calculate and recruit a sufficient sample size, the absence of any previous within-person correlation data, together with the high attrition rate, represents a limitation of this study, meaning that we may have been underpowered to detect changes in the primary burnout outcome (exhaustion). Furthermore, although the observed intervention effects for secondary outcomes are promising, the small sample size and number of between-group comparisons mean that there is a risk of type-I and type-II errors. Further research is needed to determine whether the findings of this early-phase study can be replicated in larger samples of medical students.

Intervention outcomes should also be interpreted with caution because of the potential for overestimation of effect sizes when a waitlist comparison group is used in behavior change research [106]. The waitlist group was a necessary ethical component of this study as stakeholders from the participating university medical schools wanted to ensure that all interested students were offered access to the intervention app as a well-being resource so that no students would be disadvantaged by allocation to the control condition. However, previous research suggests that waitlist participants’ expectations of receiving support at a later time could hinder the self-driven behavior change that they might otherwise have made during the same period [106]. We note that students allocated to the waiting list in this study tended not to engage with the app once they were granted access. Future replication studies might adopt an active control condition in which participants are encouraged to check in to the app regularly during the study period (eg, to complete the psychological flexibility EMA screening question without exposure to the intervention content) [75,107]. This could reduce the likelihood of disrupting any natural improvements that control group participants might make during the study period as well as facilitating subsequent access to the intervention by promoting continued engagement from the point of students’ initial interest.

Although our intervention effect analyses adjusted for participant demographic characteristics that were identified as imbalanced after randomization (gender and studying medicine as a first career), there were also slight imbalances in other factors (current therapy and year of study) that may have affected outcomes. Depending on the therapeutic approach, participation in psychological treatment while using the app could potentially support or contradict the goals of the ACT intervention. Similarly, the app might affect psychological outcomes differently during the earlier academic stages of medical education compared with the applied clinical stages. Although stratification or matched-pair sampling could have mitigated group imbalances, the functional limitations of the current app did not facilitate these approaches. Ideally, improvements in the technology could support this methodology in the future. However, using the current randomization method, future research with larger samples should resolve such imbalances and provide a clearer picture of the impact of the intervention on outcomes in heterogeneous medical student samples. Alternatively, eligibility criteria could be modified to select more homogeneous samples based on participant demographic characteristics that are identified as having the potential to affect outcomes.

The generalizability of these findings may be limited given the predominantly nonindigenous Australian sample with a slight skew toward female participants. However, research suggests that female medical students may be at greater risk of burnout [34,66], and thus, intervention studies may be particularly relevant to female participants.

### Implications and Future Directions

The aim of this RCT was to evaluate the effectiveness of individualized and nonindividualized versions of an app-delivered ACT intervention with the broader goal of contributing data that could inform future early intervention strategies for reducing burnout and improving well-being among medical students. Although we did not observe significant postintervention benefits for primary (exhaustion) or secondary (cynicism and academic efficacy) burnout outcomes, analyses of other relevant secondary outcomes provided promising preliminary support for the ACT intervention approach for medical students. We demonstrated that an ACT intervention may improve factors associated with future burnout development, including stress and well-being, as well as other important psychological health outcomes (ie, depression). These findings support previous literature highlighting the potential benefits of training medical students in psychological flexibility skills [31,61,62] and the value of further investigation of this approach. Replication and longitudinal studies are needed to further clarify the role that psychological flexibility and inflexibility interventions (eg, ACT) may play in improving well-being and mitigating the adverse impacts of stress within the medical profession, including burnout. Although we did not observe differences in efficacy between the individualized and nonindividualized versions of the app, the individualized intervention was beneficial for a greater number of outcomes. Further research addressing the methodological limitations observed in this study could progress key psychological flexibility literature goals related to individual heterogeneity and intervention precision.

Our findings further contribute to the nascent field of app-delivered psychological interventions for medical students [21] by demonstrating that a smartphone app could be used to improve important psychological health and well-being outcomes. This is also the first study to demonstrate that delivering an ACT intervention using an app could improve medical students’ psychological flexibility and inflexibility, which is an important finding given the broad relevance of these processes to psychological health and effective functioning [50]. App-delivered approaches provide medical students with an intervention that they can access in brief segments at a time and place that suits them, using a technology medium with which they are comfortable [63] and that allows them to maintain privacy [11,32,64]. They also offer stakeholders a cost-effective scalable intervention option that could reach a large number of students [39,64] while maintaining relevance to individual needs [75]. Although further research is needed to improve engagement and establish optimal individualization methods, this early implementation study provides promising support for the potential benefits of an ACT-based app in strengthening medical students’ adaptive psychological skills and improving psychological health and well-being outcomes.

Successful burnout prevention and well-being interventions likely require a combination of organizational and individual resource-building strategies [1]. Although the current app was delivered within an organizational context and supported by the universities in which it was implemented, intentional efforts were made to distance the intervention from the students’ university programs to ensure that they did not feel compelled to participate. However, increased organizational efforts to support participation in a psychological skill development app may have an important impact on normalizing and prioritizing commitment to personal well-being during the early and formative stages of a medical career [26,108]. Given the preliminary support for the benefits of this ACT intervention, future implementation studies might examine whether embedding the app into broader university-based burnout and well-being initiatives strengthens adherence and outcomes. We note that, despite the engagement challenges observed in this study, the students who participated experienced benefits across a range of key psychological health outcomes. Thus, although not necessarily a solution for all medical students, a psychological flexibility (ACT) skill training app could form part of a suite of well-being options provided by educational institutions.

## Appendix

Multimedia Appendix 1 Baseline psychological characteristics of study sample (Mean, SD) and comparisons with published reference samples (single-sample *t* tests and *P* values).

Multimedia Appendix 2 Study attrition rates for enrolled participants (N=143).

Multimedia Appendix 3 Demographic and psychological characteristics of participants lost to follow-up (LTF) and participants who completed the study; and between-group comparisons (chi-square or independent samples *t* tests and *P* values).

Multimedia Appendix 4 Intervention completion and adherence rates by study group (N=73).

Multimedia Appendix 5 Demographic and psychological characteristics of participants who met adherence criteria and participants who did not; and between-group comparisons (chi-square or independent samples *t* tests and *P* values).

Multimedia Appendix 6 CONSORT-EHEALTH checklist (V 1.6.1).

## Abbreviations

ACT: Acceptance and Commitment Training
EMA: ecological momentary assessment
JMP: Joint Medical Program
MBI-GS (S): Maslach Burnout Inventory General Survey for Students
RCT: randomized controlled trial
REDCap: Research Electronic Data Capture
