# Supplementary material for: Evaluation of an App-Delivered Psychological Flexibility Skill Training Intervention for Medical Student Burnout and Well-being: Randomized Controlled Trial
Source: JMIR Ment Health. 2023 Feb 6;10:e42566. doi: 10.2196/42566 (PMC9941904; doi:10.2196/42566)
Supplement: Multimedia Appendix 1 [file mental_v10i1e42566_app1.docx]

Multimedia Appendix 1: Baseline psychological characteristics of study sample (Mean, SD) and comparisons with published reference samples (single-sample t-tests and *P-*values)

|  | All available baseline (incl. nonrandomized participants) | | | Randomized participants (n=108) | | Comparison Samples | | | Single-sample t-test  (Randomized participants – Comparison Sample) | | |
| --- | --- | --- | --- | --- | --- | --- | --- | --- | --- | --- | --- |
| Outcome | **n** | **Mean (SD)** | | **Mean (SD)** | | **Characteristics** | **n** | **Mean (SD)** | ***df*** | **t** | ***P*** |
| Burnout |  |  | |  | |  |  |  |  |  |  |
| Exhaustion | 120 | 16.75 (7.31) | | 16.29 (7.20) | | Medical students: *burnout* ^66^ | 110 | 23.23 (4.74) | 216 | 8.42 | <.001 |
|  |  |  |  |  |  | Medical students:  *non-burnout* ^66^ | 163 | 14.96 (5.71) | 269 | 1.69 | .092 |
| Cynicism |  | 10.93 (7.13) | | 10.87 (6.92) | | Medical students: *burnout* ^66^ | 110 | 14.44 (5.59) | 216 | 4.19 | <.001 |
|  |  |  |  |  |  | Medical students:  *non-burnout* ^66^ | 163 | 7.59 (5.16) | 269 | 4.46 | <.001 |
| Academic Efficacy |  | 24.48 (6.49) | | 24.69 (6.47) | | Medical students: *burnout* ^66^ | 110 | 24.81 (5.35) | 216 | 0.15 | .881 |
|  |  |  |  |  |  | Medical students:  *non-burnout* ^66^ | 163 | 28.74 (3.21) | 269 | 6.83 | <.001 |
| Well-being | 120 | 42.70 (13.43) | | 43.03 (13.31) | | Medical students ^95^ | 2682 | 47 (12.67) | 2796 | 3.12 | .002 |
| Psychological Flexibility |  |  | |  | |  |  |  |  |  |  |
| Flexibility | 116 | 3.73 (0.76) | | 3.72 (0.78) | | General population ^88^ | 2663 | 3.83 (0.88) | 2769 | 1.28 | .201 |
| Inflexibility |  | 3.21 (0.90) | | 3.21 (0.91) | |  |  | 2.73 (0.90) |  | 5.43 | <.001 |
| Depression | 115 | 15.15 (10.14) | | 14.89 (10.25) | | General population ^90^ | 2914 | 6.34 (6.97) | 3020 | 12.27 | <.001 |
| Anxiety |  | 8.57 (8.18) | | 8.50 (8.09) | |  |  | 4.7 (4.91) |  | 7.67 | <.001 |
| Stress |  | 12.03 (10.32) | | 11.87 (10.34) | |  |  | 10.11 (7.91) |  | 2.24 | .025 |

66. Obregon M, Luo J, Shelton J, Blevins T, MacDowell M. Assessment of burnout in medical students using the Maslach Burnout Inventory-Student Survey: a cross-sectional data analysis. BMC Med Educ. 2020;20(1):376.

88. Grégoire S, Gagnon J, Lachance L, et al. Validation of the english and french versions of the multidimensional psychological flexibility inventory short form (MPFI-24). *Journal of Contextual Behavioral Science.* 2020;18:99-110.

90. Lovibond SH, Lovibond PF. *Manual for the Depression Anxiety Stress Scales (2nd ed.).* Sydney: Psychology Foundation of Australia; 1995.

95. Dyrbye LN, Harper W, Moutier C, et al. A multi-institutional study exploring the impact of positive mental health on medical students' professionalism in an era of high burnout. *Acad Med.* 2012;87(8):1024-1031.
