# Supplementary material for: Evaluation of an App-Delivered Psychological Flexibility Skill Training Intervention for Medical Student Burnout and Well-being: Randomized Controlled Trial
Source: JMIR Ment Health. 2023 Feb 6;10:e42566. doi: 10.2196/42566 (PMC9941904; doi:10.2196/42566)
Supplement: Multimedia Appendix 2 [file mental_v10i1e42566_app2.docx]

Multimedia Appendix 2: Study attrition rates for enrolled participants (N=143)

|  | n (% of enrolled) |
| --- | --- |
| *Did not complete app download/sign-up* | 18 (12.59) |
| *Did not complete T_1_ assessments* | 10 (6.99) |
| *Did not proceed to randomization* | 7 (4.90) |
| *Randomized: did not complete post assessments* | 40 (27.97) |
| *Total attrition* | 75 (52.45) |
