# Supplementary material for: Evaluation of an App-Delivered Psychological Flexibility Skill Training Intervention for Medical Student Burnout and Well-being: Randomized Controlled Trial
Source: JMIR Ment Health. 2023 Feb 6;10:e42566. doi: 10.2196/42566 (PMC9941904; doi:10.2196/42566)
Supplement: Multimedia Appendix 3 [file mental_v10i1e42566_app3.docx]

Multimedia Appendix 3: Demographic and psychological characteristics of participants lost to follow-up (LTF) and participants who completed the study; and between-group comparisons (chi-square or independent samples t-tests, and *P*-values)

| *Categorical Variables* | | Completed  (n=68) | LTF  (n=75) ^a^ | *χ^2^ (df,N)* | *P* |
| --- | --- | --- | --- | --- | --- |
|  |  | ***n*** | |  |  |
| Gender | Female | 41 | 47 | 3.34 (2,142) | .808 ^b^ |
|  | Male | 24 | 27 |  |  |
|  | Nonbinary | 3 | 0 |  |  |
| Uni | UoN | 45 | 55 | 1.13 (1,142) | .288 |
|  | UNE | 23 | 19 |  |  |
| Study Year | 1 | 21 | 23 | 0.17 (3,142) | .982 |
|  | 2 | 21 | 22 |  |  |
|  | 4 | 13 | 13 |  |  |
|  | 5 | 13 | 16 |  |  |
| Enrolment | Domestic | 64 | 71 | 0.25 (1,142) | .615 |
|  | International | 4 | 3 |  |  |
| Indigenous | Indigenous | 4 | 6 | 2.68 (1,142) | .605 |
|  | Non-indigenous | 64 | 68 |  |  |
| First career | Yes | 12 | 22 | 2.84 (1,142) | .092 |
|  | No | 56 | 52 |  |  |
| Previous Burnout | Yes | 57 | 65 | 0.47 (1,142) | .492 |
|  | No | 11 | 9 |  |  |
| Current Therapy | Yes | 15 | 15 | 0.07 (1,142) | .794 |
|  | No | 53 | 59 |  |  |
| Health | Very poor | 1 | 1 | 5.35 (4,142) | .225 ^b^ |
|  | Poor | 8 | 2 |  |  |
|  | Average | 20 | 19 |  |  |
|  | Good | 29 | 37 |  |  |
|  | Excellent | 10 | 15 |  |  |
| Diet | Very poor | 0 | 1 | 7.41 (4,142) | .098 ^b^ |
|  | Poor | 6 | 2 |  |  |
|  | Average | 18 | 29 |  |  |
|  | Good | 41 | 35 |  |  |
|  | Excellent | 3 | 7 |  |  |
| Self-care | Very poor | 0 | 1 | 1.90 (4,142) | .306 ^b^ |
|  | Poor | 12 | 15 |  |  |
|  | Average | 31 | 33 |  |  |
|  | Good | 21 | 23 |  |  |
|  | Excellent | 4 | 2 |  |  |
| *Continuous Variables* | | ***M (SD)*** | | ***t(df)*** | ***P*** |
| Age | | 24.33 (5.89) | 24.85 (5.48) | -0.37 (70) | .713 |
| Years in workforce | | 5.59 (6.37) | 6.31 (5.30) | -0.49 (70) | .626 |
| Burnout | Exhaustion | 15.87 (7.05) | 14.89 (7.56) | 0.56 (71) | .578 |
|  | Cynicism | 10.57 (6.88) | 10.56 (6.62) | 0.006 (71) | .995 |
|  | Academic Efficacy | 25.35 (6.72) | 25.48 (6.19) | -0.08 (71) | .933 |
| Wellbeing | | 3.19 (0.98) | 3.2 (0.87) | -0.05 (71) | .959 |
| Psychological Flexibility | Flexibility | 3.76 (0.77) | 3.76 (0.87) | -0.05 (71) | .624 |
|  | Inflexibility | 3.24 (0.96) | 2.99 (0.82) | 1.15 (71) | .963 |
| Psychological Distress | Depression | 12.65 (11.42) | 10.74 (9.75) | 0.73 (71) | .256 |
|  | Anxiety | 9.65 (9.06) | 6.59 (7.03) | 1.51 (71) | .469 |
|  | Stress | 16.04 (11.45) | 14.07 (9.76) | 0.75 (71) | .136 |

**^a^** Demographic data missing for n=1 in this group

**^b^** Fishers exact test
