# Supplementary material for: Evaluation of an App-Delivered Psychological Flexibility Skill Training Intervention for Medical Student Burnout and Well-being: Randomized Controlled Trial
Source: JMIR Ment Health. 2023 Feb 6;10:e42566. doi: 10.2196/42566 (PMC9941904; doi:10.2196/42566)
Supplement: Multimedia Appendix 4 [file mental_v10i1e42566_app4.docx]

Multimedia Appendix 4: Intervention completion and adherence rates by study group (N=73)

| Study group | Allocation  n | Stage 1 completed  n (% of group) | At least 1 Stage 2 skill  n (% of group) | Adherence criteria met  n (% of group) |
| --- | --- | --- | --- | --- |
| *II* | 37 | 29 (78%) | 22 (59%) | 14 (38%) |
| *NI* | 36 | 17 (47%) | 14 (39%) | 7 (19%) |
| *Total* | 73 | 46 (63%) | 36 (49%) | 21 (29%) |
