# Supplementary material for: Evaluation of an App-Delivered Psychological Flexibility Skill Training Intervention for Medical Student Burnout and Well-being: Randomized Controlled Trial
Source: JMIR Ment Health. 2023 Feb 6;10:e42566. doi: 10.2196/42566 (PMC9941904; doi:10.2196/42566)
Supplement: Multimedia Appendix 5 [file mental_v10i1e42566_app5.docx]

Multimedia Appendix 5: Demographic and psychological characteristics of participants who met adherence criteria and participants who did not; and between-group comparisons (chi-square or independent samples t-tests, and *P*-values)

| *Categorical Variables* | | Adhered  (n=21) | Did not adhere  (n=52) ^a^ | *χ^2^ (df,N)* | *P* |
| --- | --- | --- | --- | --- | --- |
|  |  | ***n*** | |  |  |
| Intervention Arm | Individualized | 14 | 23 | 3.01 (1,73) | .083 |
|  | Nonindividualized | 7 | 29 |  |  |
| Gender | Female | 13 | 30 | 0.06 (1,72) | .809 |
|  | Male | 8 | 21 |  |  |
| Uni | UoN | 16 | 33 | 0.90 (1,73) | .342 |
|  | UNE | 5 | 18 |  |  |
| Study Year | 1 | 5 | 10 | 1.97 (3,72) | .579 |
|  | 2 | 9 | 20 |  |  |
|  | 4 | 2 | 12 |  |  |
|  | 5 | 5 | 9 |  |  |
| Enrolment | Domestic | 19 | 50 | 2.13 (1,72) | .202 ^c^ |
|  | International | 2 | 1 |  |  |
| Indigenous | Indigenous | 3 | 3 | 1.38 (1,72) | .241 |
|  | Non-indigenous | 18 | 48 |  |  |
| First career | Yes | 10 | 41 | 7.73 (1,72) | .005 |
|  | No | 11 | 10 |  |  |
| Previous Burnout | Yes | 17 | 48 | 2.94 (1,72) | .183 ^c^ |
|  | No | 4 | 3 |  |  |
| Current Therapy | Yes | 2 | 11 | 1.46 (1,72) | .227 |
|  | No | 19 | 40 |  |  |
| Health | Very poor | 0 | 1 | 3.23 (4,72) | .529 ^c^ |
|  | Poor | 3 | 3 |  |  |
|  | Average | 3 | 11 |  |  |
|  | Good | 9 | 27 |  |  |
|  | Excellent | 6 | 9 |  |  |
| Diet | Very poor | 0 | 1 | 6.38 (4,72) | .113 ^c^ |
|  | Poor | 2 | 1 |  |  |
|  | Average | 2 | 17 |  |  |
|  | Good | 15 | 28 |  |  |
|  | Excellent | 2 | 4 |  |  |
| Self-care | Very poor | 0 | 1 | 8.14 (4,72) | .064 ^c^ |
|  | Poor | 3 | 9 |  |  |
|  | Average | 5 | 26 |  |  |
|  | Good | 12 | 12 |  |  |
|  | Excellent | 1 | 3 |  |  |
| *Continuous Variables* | | ***M (SD)*** | | ***t(df)*** | ***P*** |
| Age | | 27.48 (7.87) | 23.29 (4.04) | –2.31 (70) **^b^** | .029 |
| Years in workforce | | 8.29 (8.47) | 4.84 (4.30) | –1.77 (70) **^b^** | .089 |
| Burnout | Exhaustion | 15.05 (7.92) | 15.69 (6.96) | 0.34 (71) | .732 |
|  | Cynicism | 11.67 (7.50) | 10.12 (6.43) | –0.89 (71) | .377 |
|  | Academic Efficacy | 25.71 (5.81) | 25.27 (6.79) | –0.26 (71) | .793 |
| Wellbeing | |  | 3.13 (1.02) | 3.22 (0.91) | .700 |
| Psychological Flexibility | Flexibility | 3.91 (0.83) | 3.70 (0.79) | –1.015 (71) | .314 |
|  | Inflexibility | 3.23 (0.98) | 3.12 (0.90) | –0.494 (71) | .623 |
| Psychological Distress | Depression | 10.86 (11.18) | 12.38 (10.73) | 0.544 (71) | .588 |
|  | Anxiety | 9.24 (10.46) | 8.23 (7.58) | –0.459 (71) | .648 |
|  | Stress | 15.14 (12.19) | 15.38 (10.36) | 0.086 (71) | .932 |

**^a^** Demographic data missing for n=1 in this group

**^b^** Welch’s t-test conducted due to violation of Student t-test assumption of equal variance

**^c^** Fishers exact test
